# Supplementary material for: Programmable spatial magnetization stereolithographic printing of biomimetic soft machines with thin-walled structures
Source: Nat Commun. 2024 Nov 30;15:10442. doi: 10.1038/s41467-024-54773-2 (PMC11608252; doi:10.1038/s41467-024-54773-2)
Supplement: Supplementary file 1 — Supplementary Information [file 41467_2024_54773_MOESM1_ESM.pdf]

Supplementary Information

Programmable spatial magnetization stereolithographic printing of biomimetic soft machines with thin-walled structures

Xianghe Meng[1†], Shishi Li[1†], Xingjian Shen[1], Chenyao Tian[1], Liyang Mao[1], Hui Xie[1*]

[1]State Key Laboratory of Robotics and Systems, Harbin Institute of Technology, Harbin, 150080, China.

*Corresponding author(s). E-mail(s): xiehui@hit.edu.cn;
†These authors contributed equally to this work.

Supplementary Discussion

1. Generation of high-Intensity uniform 3D magnetic fields

The three-dimensional magnetic field apparatus consists of Halbach arrays embedded with electromagnet wire coils. The Halbach array generates magnetic fields parallel to the curing plane (Supplementary Fig. 1a), while the wire coils generate magnetic fields perpendicular to the curing plane (Supplementary Fig. 1b). Based on the superposition principle of magnetic vectors, the apparatus can generate a high-intensity, direction-controllable, three-dimensional uniform magnetic field.

The Halbach array's magnetic structure can be viewed as a circular ring with continuously rotating magnetization, capable of producing a constant, high-intensity, unidirectional magnetic field internally[1]. The magnetic field strength is given by:

$$B = B_{\mathrm{rem}} \cdot \ln\left(\frac{r_{\mathrm{outer}}}{r_{\mathrm{inner}}}\right) \tag{1}$$

where B_{rem} is the remanent magnetic strength of the magnetic structure, r_{outer} and r_{inner} are the outer and inner radii of the ring, respectively. Continuous variation in magnetization direction in an ideal magnetic structure cannot be practically realized. Therefore, we approximate it by discretely arranging a number of small square-shaped permanent magnets with their remanent magnetization strengths and

magnetization directions differing by small angles. Modeling an array of N point-dipoles, the magnetic field $\mathbf{B}_{\text{centre}}$ at the center of the Halbach array, is the vector sum of the fields of each dipole $\mathbf{B}_i$ as shown[2]:

$$\mathbf{B}_{\text{centre}} = \sum_{i=1}^{N} \mathbf{B}_i \mathbf{R} \tag{2}$$

where $\mathbf{R}$ is the radius vector of each of the N dipoles. The magnetic field strength of the Halbach array along the Z-axis is expressed as:

$$B_{\text{centre}}(z) = \frac{3N\mu_0 m r^2}{8\pi(r^2 + z^2)^{5/2}} \tag{3}$$

Where $\mu_0 = 4\pi \times 10^{-7}\,\mathrm{T \cdot m/A}$ is the permeability of vacuum, m is the magnetic dipole moment of each dipole, r is the radius of the dipole array, and z is the distance from the center of the Halbach array along the Z-axis.

A configuration of three Halbach arrays arranged longitudinally can generate an in-plane uniform magnetic field with any desired strength and direction on the curing plane, while maintaining lower rotational inertia and body mass.The upper and lower arrays rotate simultaneously, resulting in an equivalent magnetic field $\mathbf{B}_{\text{equivalent}}$, while the middle array generates a magnetic field $\mathbf{B}_{\text{middle}}$. The combination of these two magnetic fields gives the total magnetic field $\mathbf{B}_{\text{total}}$:

$$\mathbf{B}_{\text{total}} = \mathbf{B}_{\text{equivalent}} + \mathbf{B}_{\text{middle}} \tag{4}$$

Only when $|\mathbf{B}_{\text{equivalent}}| = |\mathbf{B}_{\text{middle}}|$, the Halbach array combination can achieve a uniform magnetic field strength by adjusting the rotation angle α (the angle between adjacent Halbach arrays), generating a $\mathbf{B}_{\text{total}}$ with the desired magnitude. The magnetic field magnitude is given by:

$$|\mathbf{B}_{\text{total}}| = |\mathbf{B}_{\text{equivalent}}| \cdot \cos\alpha + |\mathbf{B}_{\text{middle}}| \cdot \cos\alpha \tag{5}$$

Using three-dimensional Hall probes to measure the magnetic field, the Halbach array combination can produce a magnetic field strength B_y at the center of the curing plane: $[-83.3\text{mT}, 83.5\text{mT}]$. The magnetic field strength fluctuates with the angle α. The error components in the other two directions are B_x and B_z, given by $[-1.6\text{mT}, 1.7\text{mT}]$ and $[-0.2\text{mT}, 0.2\text{mT}]$ respectively. Among them, the error component in the B_x direction is within 2% of the maximum magnetic field strength, which is acceptable and meets the design requirements.

In practice, $|\mathbf{B}_{\text{equivalent}}| \neq |\mathbf{B}_{\text{middle}}|$, resulting in an incomplete superposition of the magnetic fields. However, this deviation is relatively small and does not have a noticeable effect.

The electromagnetic solenoid can generate a uniform magnetic field perpendicular to the curing plane, which can be simplified as multiple superimposed current-carrying circular coils. According to the Biot-Savart law, the magnetic field strength $B(z)$ at a distance z from the wire is given by:

$$B(z) = \frac{\mu_0 I r^2}{2(r^2 + z^2)^{3/2}} \tag{6}$$

where μ_0 is the permeability of free space, I is the current, and r is the radius of the wire loop.

For a solenoid with multiple turns, the magnetic field strength along the axis inside the solenoid is:

$$B = \int_{z_1}^{z_2} \frac{\mu_0 n I r^2}{2(r^2 + z^2)^{3/2}}\, dx \tag{7}$$

where z_1 and z_2 are the ends of the solenoid, n is the number of turns per unit length, and I is the current in the wire.

Measurements show that the solenoid can produce a magnetic field strength at the center of the curing plane B_z in the range of $[-81.0\text{mT}, 81.3\text{mT}]$, linearly related to the input current $[-10\text{A}, 10\text{A}]$. The error components are B_x and B_y, given by $[1.4\text{mT}, 1.6\text{mT}]$ and $[-0.7\text{mT}, 0.7\text{mT}]$ respectively. The error components in the B_x and B_y direction are within 2% of the maximum magnetic field strength.

2. Magnetic field uniformity and directional accuracy in printing area

Using three-dimensional Hall probes, we measured the 80mT magnetic field vector's three-dimensional distribution and angular error at the center of the curing plane (Supplementary Fig. 2a). We set θ from $0°$ to $180°$ and φ from $0°$ to $360°$, both with $30°$ intervals. The angular error at the center ranged from $0.1°$ to $1.3°$ compared to theoretical values. The error was larger when the magnetic field direction approached the normal to the curing plane. This is because, as the normal component increases, the in-plane component decreases, increasing the angle between the Halbach arrays. Since the magnetic field generated by the Halbach arrays cannot be fully zeroed, there is a fluctuation within 2% of the maximum field strength, leading to larger errors when the magnetic field is near the normal direction. The measured magnetic field strength ranged from 78.7 mT to 81.0 mT, with an error within 2% of the theoretical value.

We characterized the uniformity of the magnetic field strength and direction over a 40mm diameter printing area using both simulation calculations and three-dimensional magnetometer measurements (Fig. 1e and Supplementary Fig. 2b). First, we set the magnetic field to $(80\text{mT}, 90°, 0°)$, generated by the Halbach arrays. The measured values were taken from 5 points evenly spaced 10mm apart along the X and Y axes. The observed magnetic field strength ranged from 80.0 mT to 83.4 mT, with a non-uniformity of less than 5%, and the maximum angular error was about $1°$. The simulation data covered the entire printing area and showed a magnetic field strength range of 82.3mT to 85.9mT, with a non-uniformity of less than 5%. The magnetic field vectors, represented by arrows in the plane, demonstrated good directional consistency. The simulation data closely matched the measured values, confirming their reliability.

Next, we set the magnetic field to $(80\text{mT}, 0°, 0°)$, generated by an electromagnetic solenoid. Simulation calculations showed that the magnetic field strength across the entire printing area ranged from 79.8 mT to 81.9 mT, with a non-uniformity of less than 3%, and the magnetic field direction exhibited good consistency. Compared to the in-plane magnetic field parallel to the curing plane, the normal magnetic field perpendicular to the curing plane had better uniformity and directional accuracy. This is because the uniformity of the magnetic field generated by the Halbach arrays is lower compared to that generated by the electromagnetic solenoid. Therefore, when the in-plane magnetic vector component is larger, the magnetic field uniformity decreases.

3. Magnetic field uniformity analysis and simulation validation

Fig. 1e shows a discrepancy between simulated and measured magnetic field data. The simulation data within the 40mm diameter printable range showed a field strength range of 82.3 mT to 85.4 mT and an angle error range of $0°$ to $0.7°$. Measurements at 10 mm intervals along the X and Y axes revealed a field strength range of 80.0 mT to 83.4 mT and an angle error range of $0.1°$ to $0.9°$.

The measured magnetic field strength was consistently lower by approximately 2 mT compared to the simulation, while maintaining a similar curve shape. Simulated magnetic field angle errors were generally smaller than measured values.

To address this discrepancy, the simulation's material properties were recalibrated. The remanent flux density was adjusted from 1.44 T to 1.40 T. The updated simulation results showed a magnetic field strength range of 80.0 mT to 83.4 mT (Supplementary Fig. 20a) and an angle error range of $0°$ to $0.4°$ (Supplementary Fig. 20b).

The revised simulation results align more closely with the measured data in both trends and ranges, particularly in magnetic field strength. This alignment supports the main conclusions in the primary manuscript and suggests that the observed discrepancy primarily resulted from a mismatch between the actual permanent magnets' properties and the initial simulation parameters.

4. Effect of different dispersion conditions on the stability of printing materials

We prepared four printing materials with different dispersion compositions: G1 (10 wt% ground 1 − 2 μm NdFeB + 2 wt% fumed silica), G2 (10 wt% commercial 5 μm NdFeB + 2 wt% fumed silica), G3 (10 wt% ground 1 − 2 μm NdFeB), and G4 (10 wt% commercial 5 μm NdFeB). Using a spectrophotometer (721, Jinghua Instruments), we measured absorbance (Fig. 2c) and observed the physical images (Supplementary Fig. 4) to study the effect of different dispersion conditions on the stability of printing materials under an external magnetic field of 80 mT.

A planetary mixer is used to mix the printing materials, ensuring uniform dispersion of magnetic particles in the matrix and forming a colloidal suspension. The printing material is transferred to a cuvette for measurement using a spectrophotometer. The spectrophotometer is preheated for 30 minutes to maintain stable operation. The absorbance of the printing material depends on the concentration of the absorbing species, the magnetic particles. Therefore, the sedimentation of magnetic particles in the matrix can be characterized by changes in absorbance. Simultaneously, as sedimentation occurs, magnetic particles and the printing material may show stratification, observable through physical images.

By monitoring changes in absorbance and physical appearance, we observe significant differences in the stability of the printing materials under the influence of an 80 mT external magnetic field over an 8-hour period. G1 maintains a stable suspension for 8 hours, with only a slight 2% decrease in absorbance and no stratification observed. G2 maintains stability for 4 hours, with an 8-hour observation showing a significant decrease in absorbance due to the sedimentation of magnetic particles. G3 only maintains stability for 15 minutes, with rapid aggregation of magnetic particles, and G4 fails to maintain stability with rapid sedimentation and stratification observed shortly after mixing.

5. Effect of curing time on the curing depth of printing materials

Based on the Beer-Lambert Law, the absorbance of the printing material is determined by the concentration of the absorbing species, which include magnetic particles and photoinitiator[3]. The intensity at a depth z is expressed as:

$$I(z) = I_0 e^{-\mu z} \tag{8}$$

where I_0 is the intensity at $z = 0$ (the surface closest to the UV light source), $\mu = \ln 10(\epsilon_m c_m + \epsilon_i c_i)$, ϵ_m and c_m are the molar absorptivity and concentration of the NdFeB particles, and ϵ_i and c_i are the molar absorptivity and concentration of the photoinitiator. Assuming the threshold energy required for curing the printing material is E_c, and the minimum time required to start curing at $z = 0$ is t_0, I_0 can be expressed as:

$$I_0 = \frac{E_c}{t_0} \tag{9}$$

The intensity I_z at depth z must reach the threshold energy E_c, with an exposure time t_z, i.e.:

$$I_z = \frac{E_c}{t_z} \tag{10}$$

Thus, the relationship between the curing depth z and exposure time t_z can be derived as:

$$z = \frac{\ln\left(\frac{t_z}{t_0}\right)}{\mu} \tag{11}$$

The curing depth is linearly related to the logarithm of the exposure time. Measurements of the curing depth for printing materials with NdFeB particles at 10 wt%, 15 wt%, and 20 wt%, with exposure times ranging from 10 to 40 s, are shown in Supplementary Fig. 5. Observations indicate that with the increase in the concentration of NdFeB particles, which are the main absorbing species, the maximum curing depth decreases. As the exposure time increases, the curing depth trends towards a constant value at around 40s. The results align with theoretical predictions.

6. Layer thickness uniformity analysis

The uniformity of layer thickness in 3D-printed structures was analyzed to address potential variations between edge and middle parts. This non-uniformity is primarily attributed to the Gaussian light intensity distribution of individual pixels in the DLP projector[4]. To quantify this effect, additional analyses were conducted using a sample with 200 μm layer thickness and 10 wt% NdFeB content.

Investigation revealed that the actual curing depth was 240 μm, which is 1.2 times the set layer thickness. This intentional design choice ensures sufficient cross-linking quality between layers, contributing to the structural integrity of the print despite the non-uniform thickness. At the 200 μm thickness interface, the cross-linking length at the edge (0 μm position) was initially 83% of the center.

To improve uniformity, secondary exposure compensation was implemented in the non-uniform regions. This technique enhanced the cross-linking length ratio from 83% to 95%, as evidenced by experimental results. Supplementary Fig. 21 presents a side-wall image of the cured structure and its profile curve, demonstrating the thickness variation before and after compensation.

The observed non-uniformity did not significantly impact the functionality or performance of the printed structures. While the surface quality of the printed structures showed visible layer lines, this did not affect the overall performance for the intended applications. For applications requiring higher uniformity, multiple exposures could potentially be employed to further refine the printing process[5].

7. Fluid pressure-controlled valve system in heart-inspired pump

The aortic valve-inspired one-way valve in the heart-inspired pump demonstration is designed to be controlled through two methods: switching of external magnetic fields and fluid pressure. In the demonstration, the focus was specifically on the fluid pressure control mechanism, which closely mimics the functioning of the human aortic valve. Both valves in this setup are strategically placed outside the magnetic field area, allowing their opening and closing states to be governed solely by fluid dynamics.

The operation principle of the valve is based on the response of its leaflets to fluid pressure. When fluid pressure is applied in a specific direction, the valve leaflets fold inward, creating a trifoliate opening that permits fluid passage. Conversely, when the fluid pressure is reversed, the leaflets open outward, with their edges constraining each other, effectively closing the opening and preventing fluid passage. This mechanism is illustrated in Supplementary Fig. 22a. To provide a comprehensive understanding of the valve's performance, additional experiments were conducted using an injection pump to control fluid flow through the valve at speeds ranging from 0 to 0.6 mL/s. This range was chosen to effectively cover the actual flow rate range of the heart-inspired pump (0.30 mL/s to 0.56 mL/s), ensuring that the observations are relevant to the pump's operating conditions. Using a micro-camera, cross-sectional images of the valve under various flow conditions were captured. The results, presented in Supplementary Fig. 22b, demonstrate that the valve opens at a low flow rate of 0.2 mL/s, and its opening area gradually enlarges as the flow rate increases.

The coordinated working state of the two valves during a complete pumping cycle is depicted in Supplementary Fig. 22c. The valves are installed in opposite directions to ensure unidirectional fluid flow. During the diastolic phase, as the pressure in the pump cavity decreases, liquid flows in from the outside. The left valve opens to allow fluid passage, while the right valve closes to prevent backflow from the simulated vascular network channel. In the subsequent systolic phase, the increased pressure in the pump cavity creates fluid pressure in the opposite direction. This causes the left valve to close and the right valve to open, effectively pumping fluid into the channel.

Through this coordinated operation of the two valves, efficient unidirectional fluid delivery is achieved in the heart-inspired pump demonstration.

8. Independent control of multiple capsule robots

This experiment explores the challenge of independently controlling multiple magnetic capsules and demonstrates initial progress in multi-region targeted drug delivery. The approach achieves a degree of

independent control through precise magnetization angle programming. Three capsules were manufactured with different net magnetic moments by programming their magnetization angles. The net magnetic moment of each capsule is given by $|\mathbf{M}_{\text{net}}| = |\mathbf{M}| \times \cos\alpha$, where α is half the angle between magnetization directions. The angles were set to 75°, 65°, and 55° for Capsules 1, 2, and 3 respectively, resulting in increasing radial net magnetic moments: $|\mathbf{M}_{\text{net1}}| < |\mathbf{M}_{\text{net2}}| < |\mathbf{M}_{\text{net3}}|$ (Supplementary Fig. 23a). The experiment consisted of three stages, each targeting a specific capsule (Supplementary Fig. 23b):

(i) A 5 mT, 1 Hz rotating magnetic field was applied to control Capsule 3 (which has the largest net magnetic moment). This capsule was moved to its target position, activated to release fluid simulating drug delivery, and then removed from the environment. The other two capsules remained stationary due to insufficient magnetic torque.

(ii) An 8 mT, 1 Hz rotating magnetic field was applied to control Capsule 2 (which has a medium net magnetic moment), repeating the same process, while Capsule 1 remained stationary.

(iii) A 15 mT, 0.2 Hz magnetic field was applied to control Capsule 1 (which has the smallest net magnetic moment), repeating the same process.

This experiment demonstrated the independent control of multiple capsules with different magnetic properties, selective activation of capsules using varying field strengths, and sequential, multi-region drug release simulation in an ideal environment. The ability to program different magnetic responses in multiple capsules allows for complex, targeted drug delivery strategies using a single external control system.

However, it is important to acknowledge the limitations of this approach, particularly in complex real-world applications. While these results show promise, achieving fully independent control of multiple units through a single external input remains a significant challenge. Future designs of heterogeneous robots will continue to explore methods for achieving more refined control of each capsule. While fully independent control of multiple units through a single external input remains a significant challenge, our preliminary research lays the foundation for future precise drug release at multiple target points. However, we recognize that achieving this goal in complex real-world applications will require further innovations and breakthroughs.

9. Structural optimization for reducing actuating magnetic field

An optimized structural design was developed using a thin-walled 3D structure manufacturing method. This method focuses on reducing wall thickness in areas of high strain concentration and increasing thickness in regions of lower stress to enhance the driving force. Three different characteristic structures were evaluated:

- Type 1: Cylinder with an outer diameter of 6 mm, wall thickness of 500 μm, and a height of 5 mm.
- Type 2: Wall thickness uniformly reduced to 200 μm.
- Type 3: Wall thickness of 200 μm in strain concentration areas, with an additional 300 μm thick structure in regions of lower strain.

Simulations and experiments revealed significant differences in deformation across the structures. Under a 40 mT magnetic field, the cross-sectional areas after deformation decreased by 5%, 43%, and 56% for Types 1, 2, and 3, respectively, compared to their undeformed state (Supplementary Fig. 24a). To further investigate performance, magnetic fields ranging from 20 mT to 100 mT (in 20 mT increments) were applied, and the resulting deformations were observed (Supplementary Fig. 24b). The cross-sectional deformations showed that each structure required different driving field strengths to achieve similar deformations. The contraction rate, defined as (volume before deformation - volume after deformation) / volume before deformation, was analyzed for each structure type across the range of applied magnetic fields (Supplementary Fig. 24c). This analysis demonstrated that the optimized structures reduced the required driving field strength by up to 20%, while achieving the same contraction rate, effectively lowering the required magnetic field.

These findings show that the Type 3 structure is the most efficient in minimizing the magnetic field strength needed while maintaining high deformation capability. The optimized design strategy - reducing

wall thickness in strain concentration areas and increasing it in low-stress regions - enhances driving force and reduces deformation resistance.

10. Support structure removal process

The support structure removal process employs a systematic approach consisting of cutting off supports, checking connections, and pulling out the support material (Supplementary Fig. 19a). This procedure utilizes elongated precision scissors for manual removal, with the operator relying on tactile feedback to accurately control the cutting process. Following the initial cutting, a 1.5 mm diameter semi-circular slender rod verifies the complete disconnection of supports by carefully moving along the edges. Once all connections are confirmed severed, the support structure can be safely separated from the thin-wall structure. This process is demonstrated for a complex structure in Supplementary Fig. 19b, where the support removal for a heart-inspired pump is shown step by step.

This methodology is effective for both open and semi-enclosed elastomeric printed structures, including complex 3D designs. The process leverages the passive deformation capability of the thin-wall structure post support removal, facilitating the trimming of residual supports. As observed in microscopic images (Fig. 3e), areas connected to supports show a slight thickness increase, while unconnected areas maintain the designed thickness. Residual supports shorter than 500 μm are primarily distributed in non-strain-concentrated areas of the thin-wall structure (wall thickness 200 μm-600 μm).

The presence of residual supports affects the structure minimally. While support connection points show localized increased thickness, strain-concentrated areas crucial for functionality retain their designed dimensions. Notably, these residual supports serve a beneficial purpose as additional magnetic structures, providing extra magnetic torque (Fig. 3b) without compromising essential structural characteristics.

Supplementary Figures

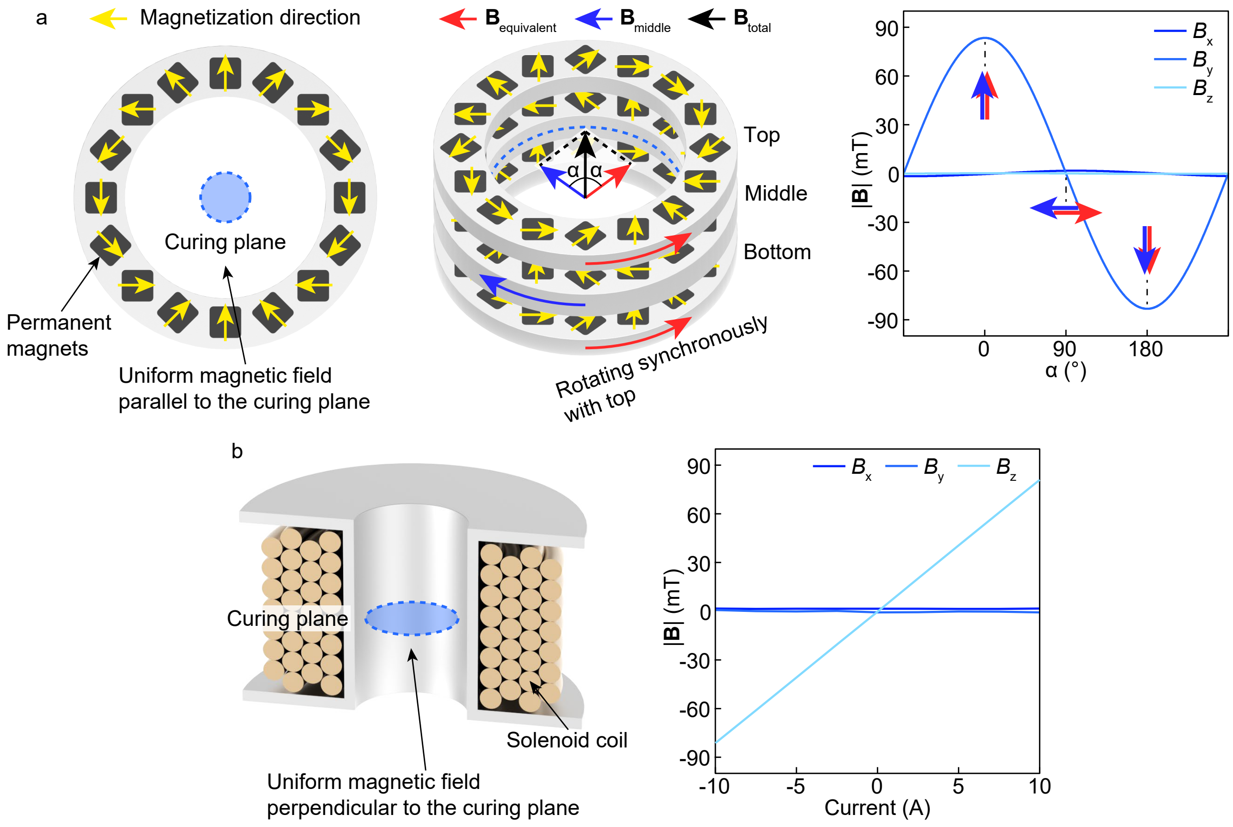

Supplementary Figure 1 Magnetic fields at the curing plane generated by Halbach arrays and an electromagnetic solenoid. (a) Halbach arrays producing an in-plane magnetic field: (left) schematic of permanent magnets creating a uniform magnetic field parallel to the curing plane, (middle) synchronous rotation of top, middle, and bottom layers showing equivalent ($\mathbf{B}_{\mathrm{equivalent}}$), middle ($\mathbf{B}_{\mathrm{middle}}$), and total ($\mathbf{B}_{\mathrm{total}}$) magnetic fields, (right) measured magnetic field components (B_{x}, B_{y}, B_{z}) vs. rotation angle α. (b) Electromagnetic solenoid generating a normal magnetic field: (left) schematic of solenoid coil producing a uniform magnetic field perpendicular to the curing plane, (right) measured magnetic field components (B_{x}, B_{y}, B_{z}) vs. current I.

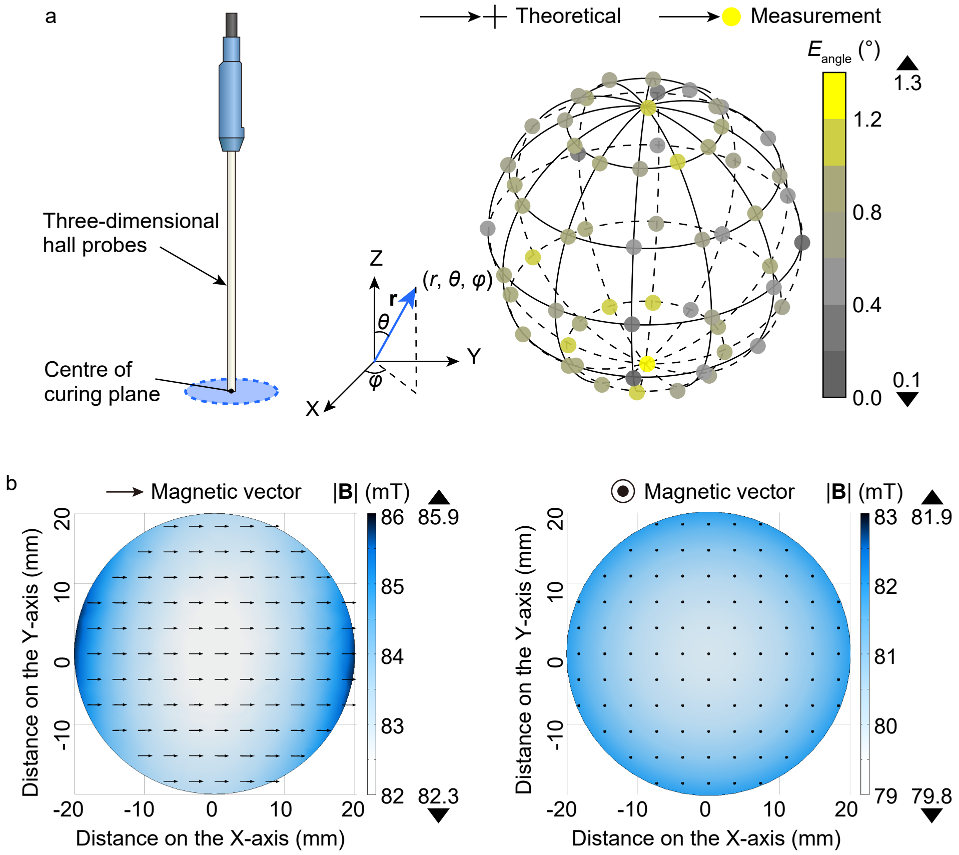

Supplementary Figure 2 Accuracy and uniformity of 3D magnetic fields in the printing plane. (a) Measurement setup using three-dimensional Hall probes at the center of the curing plane to measure an arbitrary 80 mT magnetic field direction, with a three-dimensional distribution of the magnetic field vectors and angle errors. (b) Simulation of the uniformity of magnetic field strength and direction within a 40 mm diameter printing area: (left) magnetic vector distribution and field strength ($|\mathbf{B}|$) from 82.3 to 86 mT, (right) magnetic vector distribution and field strength ($|\mathbf{B}|$) from 79.8 to 83 mT.

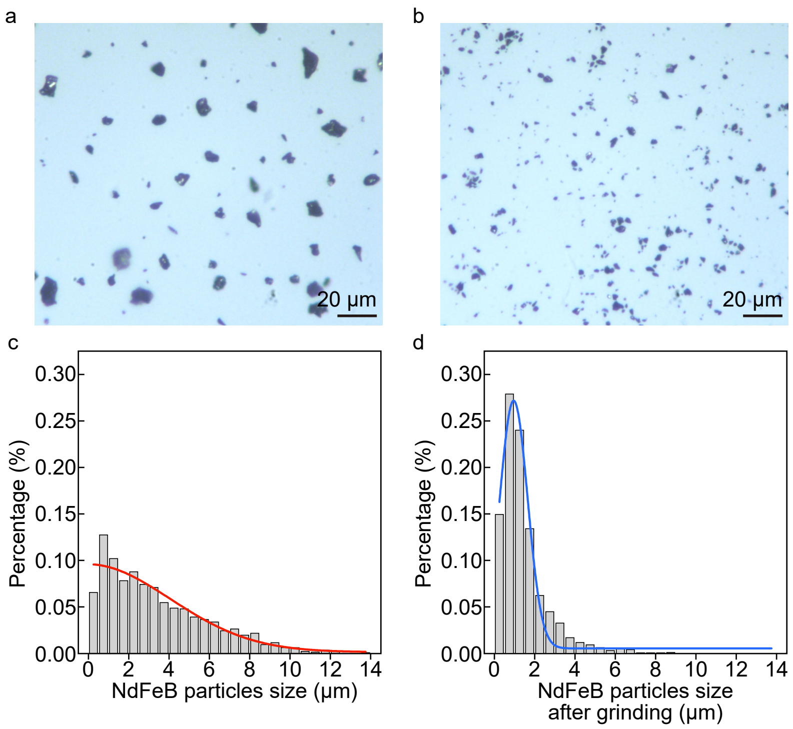

Supplementary Figure 3 Microscopy images and size distribution of NdFeB particles before and after grinding. (a) Microscopy image of commercial 5 μm NdFeB particles. (b) Microscopy image of 1-2 μm NdFeB particles after 6 hours of grinding using a ball mill. (c) Size distribution of commercial 5 μm NdFeB particles, with 75.7% having a size of $\leq$ 5μm. (d) Size distribution of ground 1-2 μm NdFeB particles, with 80.2% having a size of $\leq$ 2μm.

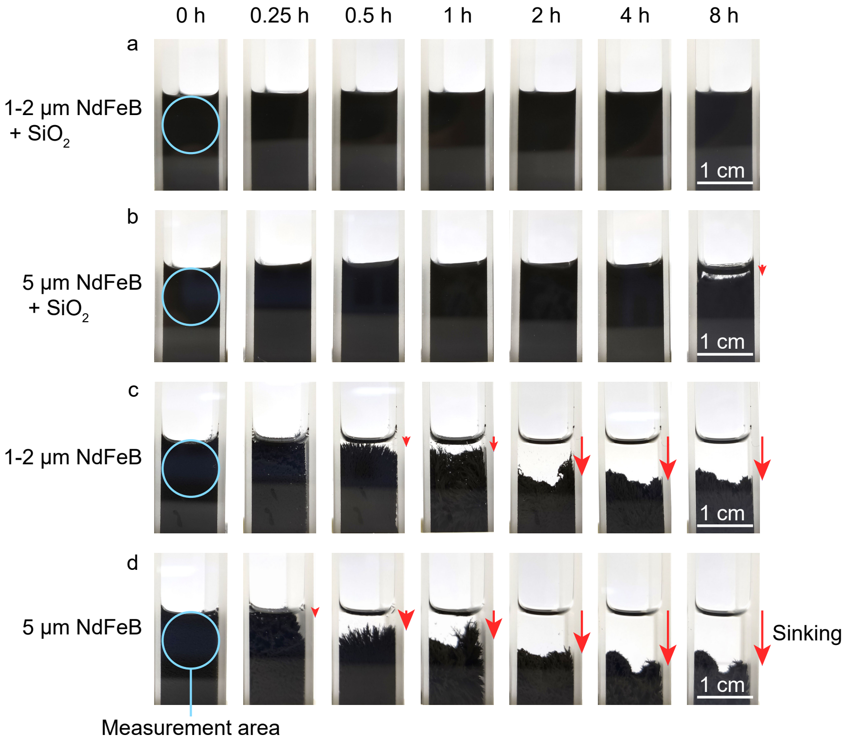

Supplementary Figure 4 Stability of NdFeB particles dispersed in resin under an 80 mT magnetic field over 8 hours. (a) G1: 10 wt% ground 1-2 µm NdFeB + 2 wt% fumed silica. (b) G2: 10 wt% commercial 5 µm NdFeB + 2 wt% fumed silica. (c) G3: 10 wt% ground 1-2 µm NdFeB. (d) G4: 10 wt% commercial 5 µm NdFeB. Each row shows the time progression from 0 to 8 hours, highlighting the sedimentation behavior and dispersion stability of the different samples.

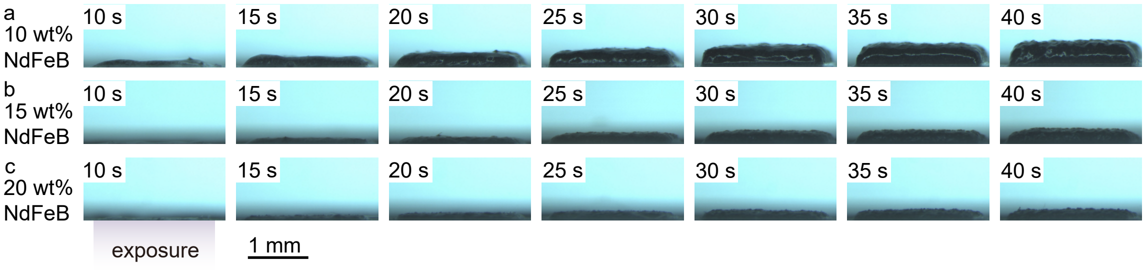

Supplementary Figure 5 Curing depth of printed materials with 10-20 wt% NdFeB under different exposure times. (a) 10 wt% NdFeB. (b) 15 wt% NdFeB. (c) 20 wt% NdFeB. Each row shows the progression of curing depth with increasing exposure time from 10 to 40 seconds.

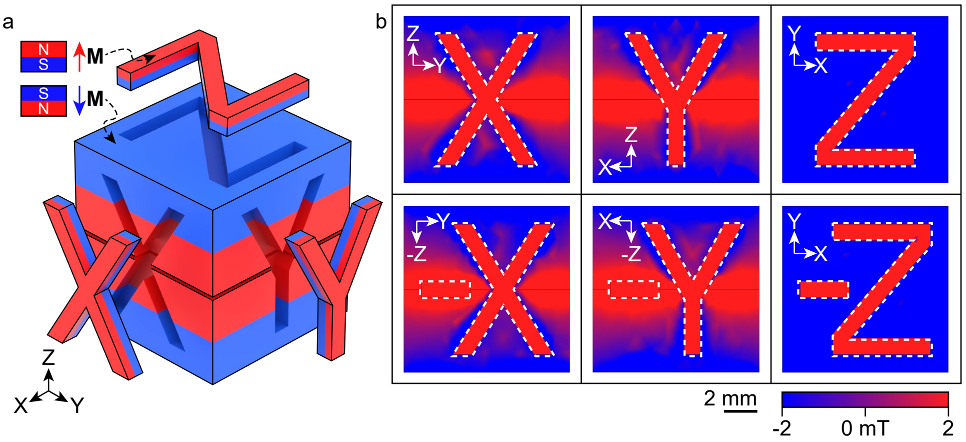

Supplementary Figure 6 Demonstration of spatial magnetization programming in a 3D printed cube structure. (a) Schematic diagram of the 3D printed cube with programmed magnetization patterns on each face. (b) COMSOL simulation results showing the magnetic field distribution on each face of the cube. The letters X, Y, and Z indicate the magnetization direction normal to each face. Dashed white lines outline the programmed letter patterns.

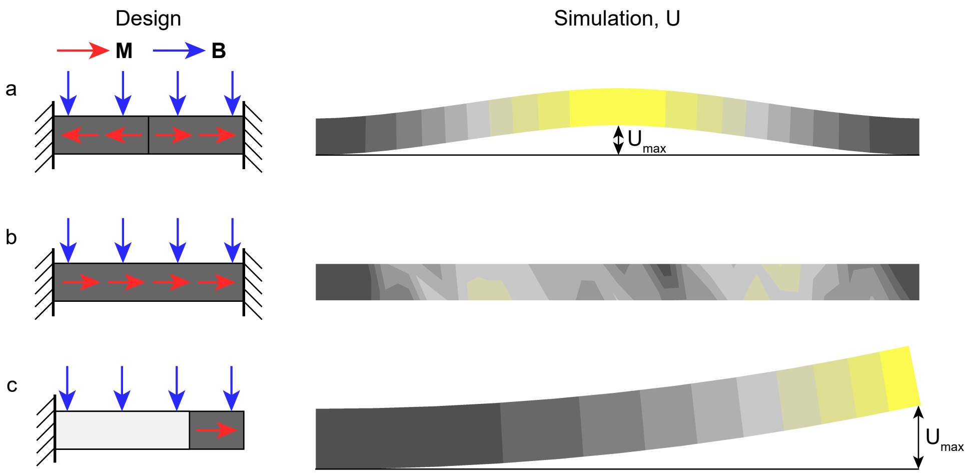

Supplementary Figure 7 Simulation of deformation in magnetic and supporting structures under an 80 mT magnetic field. (a) Magnetic structure with opposing magnetization directions fixed at both ends, showing maximum deformation at the junction where magnetization direction changes. (b) Magnetic structure with uniform magnetization direction fixed at both ends, showing no significant deformation. (c) Cantilever support structure fixed at one end and free at the other, with the free end connected to the magnetic structure, showing maximum deformation at the free end.

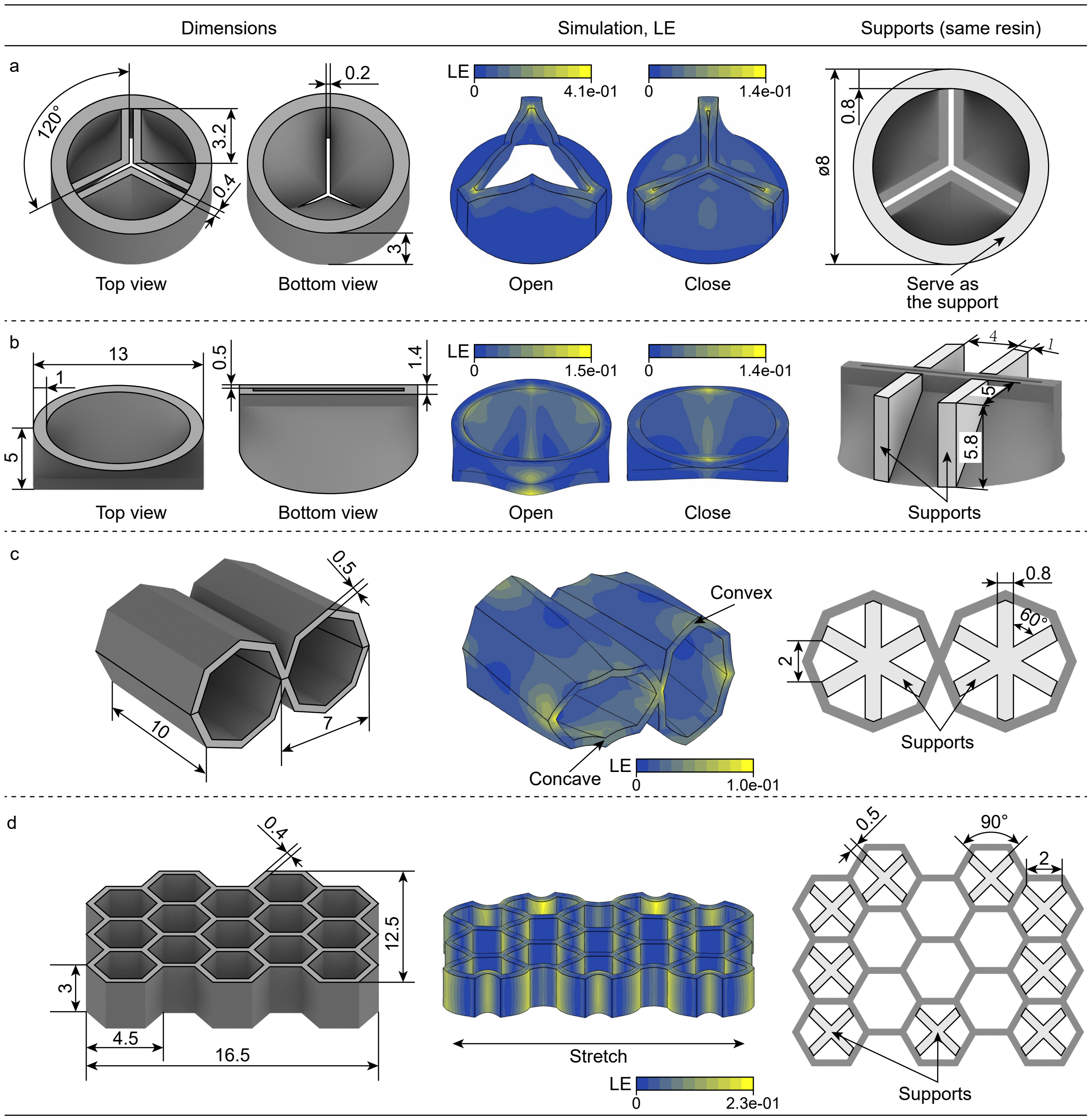

Supplementary Figure 8 Dimensions, strain simulation, and supports of printed structures in Fig. 4. (a) Biomimetic arterial valve model, showing open and closed states under alternating magnetic fields. The valve ring structure serves as support. (b) Biomimetic mitral valve model, showing open and closed states under alternating magnetic fields. The support structures are positioned around the valve ring. (c) Adjacent octagonal tube structures forming concave and convex surfaces under an external magnetic field. Support structures are added to avoid strain concentration areas. (d) Multi-cellular honeycomb structure showing uniaxial stretching under an external magnetic field. Supports are distributed only in the peripheral units, with internal units supporting each other. The dimensions in this figure are given in millimeters. Yellow highlights indicate strain concentration areas in the simulation results, where supports should be avoided.

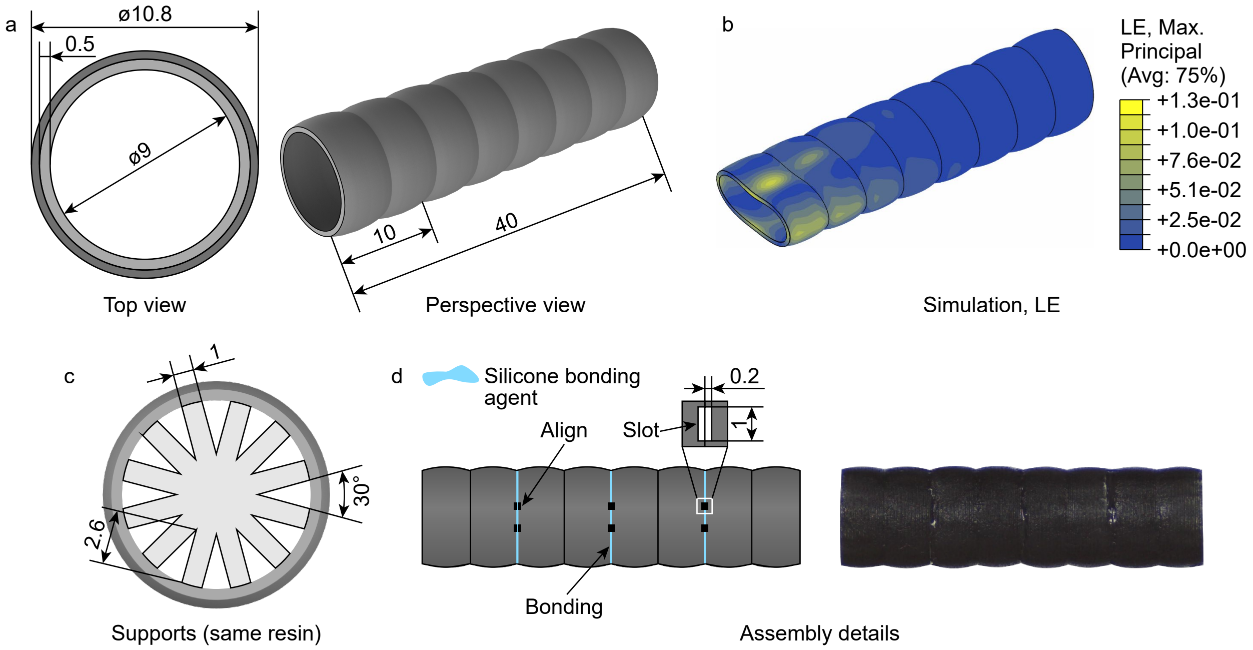

Supplementary Figure 9 Design, strain distribution, support addition, and assembly details of the peristaltic machine in Fig. 5. (a) Dimensions of the biomimetic intestinal extrusion model. (b) Strain distribution simulation of partial structure deformation under regional magnetic field actuation. (c) Regions for adding supports, including spacing and shape of supports. (d) The machine is assembled from four 10 mm long segments bonded with silicone, manufactured using parallel printing to double production efficiency. Alignment points are designed for easy assembly.

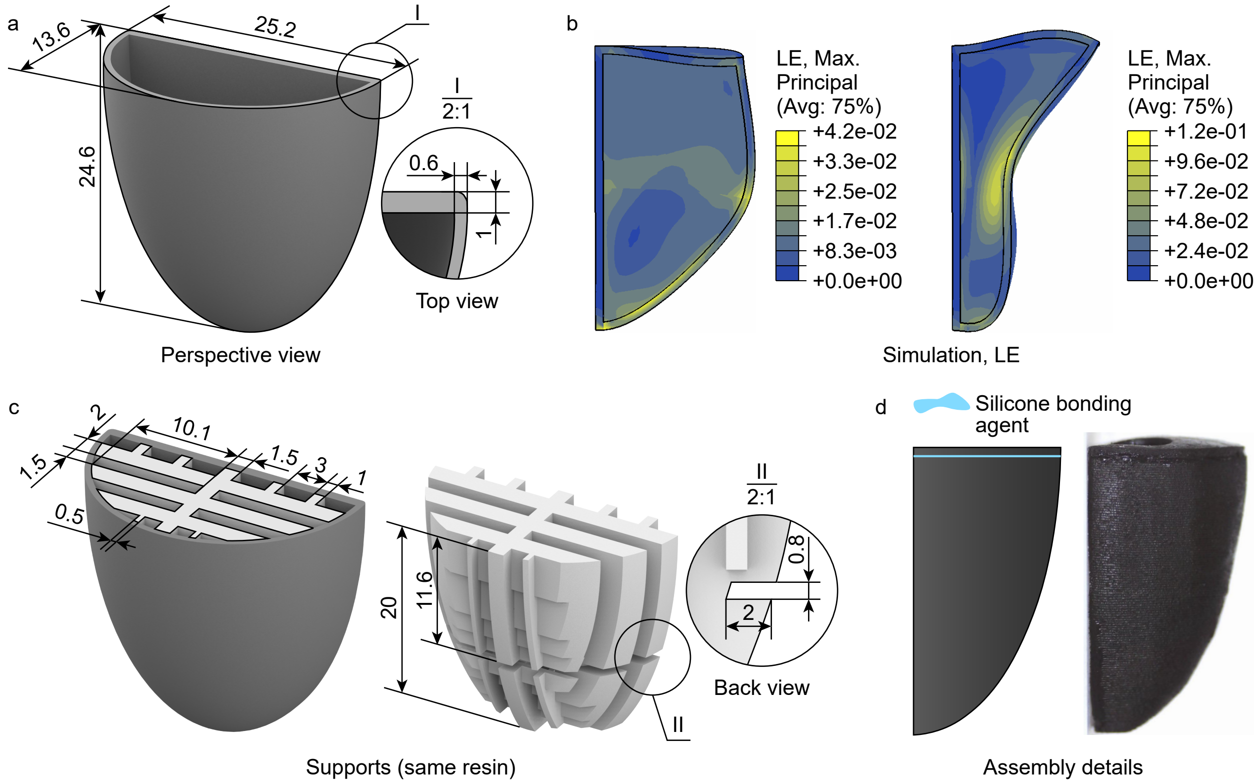

Supplementary Figure 10 Design, strain distribution, support addition, and assembly details of the soft pumping machine in Fig. 6. (a) Dimensions of the biomimetic ventricular pumping model. (b) Strain distribution simulation of the model during diastole and systole under alternating magnetic field actuation. (c) Regions for adding supports, including spacing and shape of supports. (d) Assembly detail showing a zero-magnetic-moment thin film bonded to the model's opening with silicone, creating a closed cavity structure to enable fluid control through deformation-induced pressure differentials.

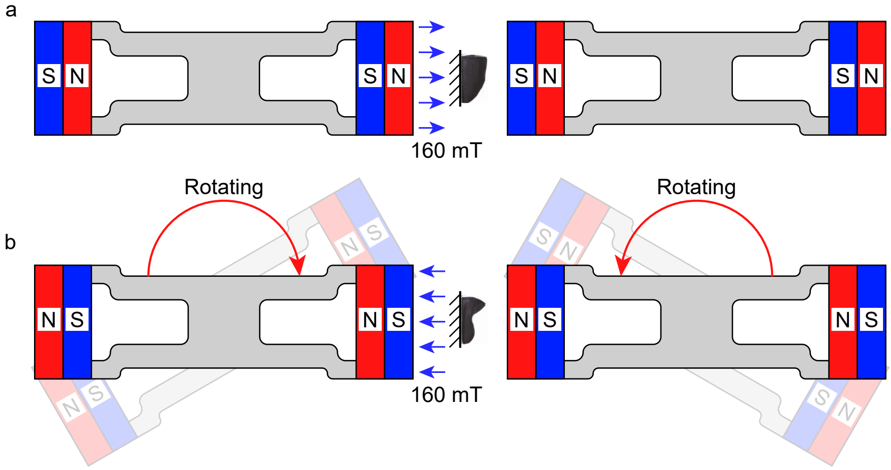

Supplementary Figure 11 Device for generating an alternating magnetic field. (a) Permanent magnets generate a leftward driving magnetic field, controlling the ventricle-inspired pump's diastolic deformation. (b) The two pairs of permanent magnets rotate 180° in opposite directions to generate a rightward driving magnetic field, controlling the ventricle-inspired pump's systolic deformation. Two pairs of opposing permanent magnets periodically rotate 180° to create an alternating magnetic field. The design includes a large 20 cm switching radius to prevent sudden changes in the magnetic field direction near the soft pump, avoiding disturbance deformation during the magnet switching process. The switching time is set to 0.75 s.

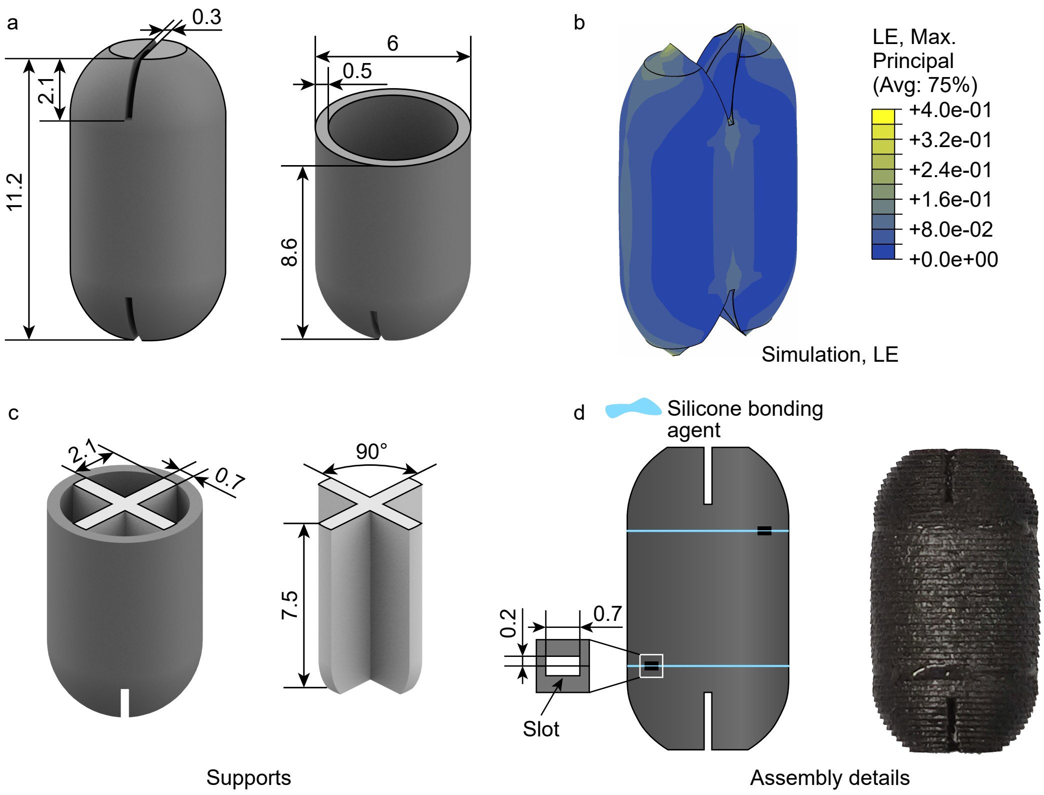

Supplementary Figure 12 Design, strain distribution, support addition, and assembly details of the magnetic capsules in Fig. 7. (a) Dimensions of the capsule. (b) Strain distribution simulation of the activated state under an external magnetic field. (c) Regions for adding supports, including spacing and shape of supports. (d) The magnetic capsule is composed of a cylindrical body and two hemispherical ends. Alignment points are designed for accurate assembly, which is achieved by bonding with silicone.

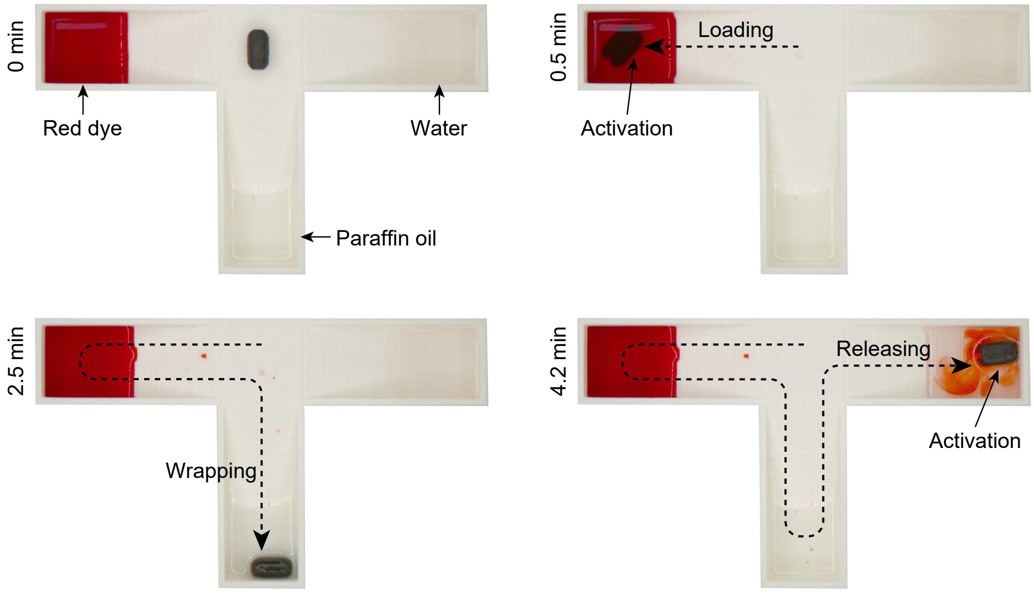

Supplementary Figure 13 Process of loading, sealing, and releasing cargo by the soft capsule robot. At 0 min, the capsule is positioned in the middle of the T-shaped path with red dye on the left, water on the right, and paraffin oil in the bottom chamber. At 0.5 min, the machine rolls into the loading chamber containing red dye under a rotating field of 30 mT at 0.1 Hz and begins activation, compressing its cavity to draw in the dye under a 200 mT field provided by a permanent magnet. At 2.5 min, the capsule, now loaded with dye, rolls uphill, breaking the solid-liquid interface, and enters the sealing chamber containing paraffin oil, where it rolls to coat itself with an oil film. At 4.2 min, the machine reaches the release chamber containing water and releases the internal dye upon activation.

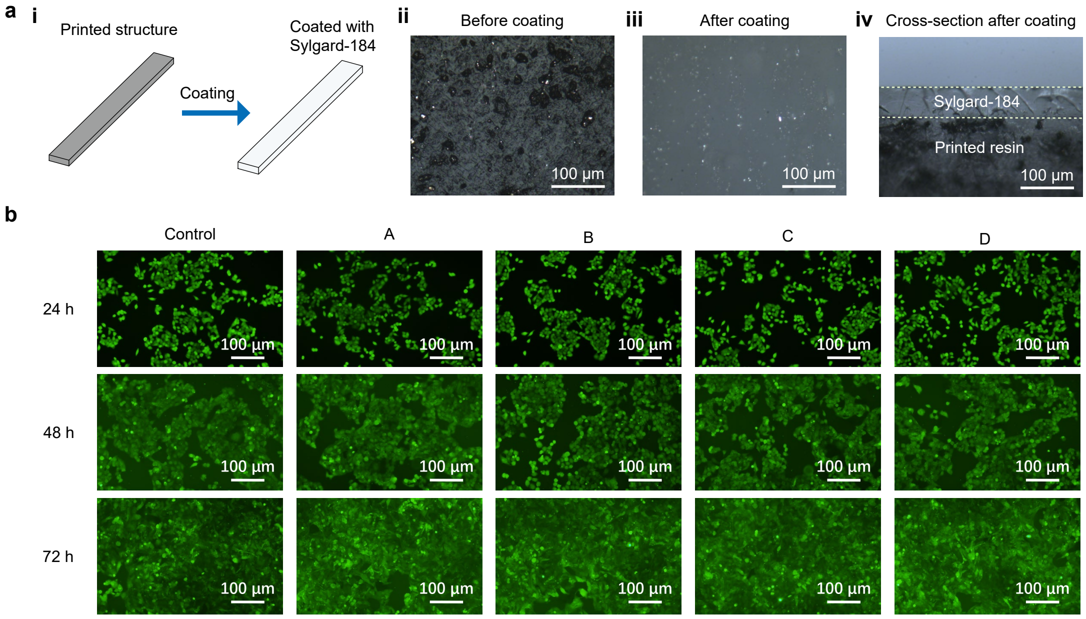

Supplementary Figure 14 Cytotoxicity test of 3D-printed material coated with Sylgard-184 (a) Surface characterization of 3D-printed material coated with Sylgard-184. (i) Schematic of the coating process of 3D-printed structures with Sylgard-184. (ii) Microscopic image of the printed material surface before coating. (iii) Microscopic image of the printed material surface after coating with Sylgard-184. (iv) Cross-sectional view after coating, showing the interface between the Sylgard-184 layer and the printed resin. (b) Cytotoxicity test results of 3D-printed material coated with Sylgard-184. Control group (Control) and different treatment groups (A: pure Sylgard-184; B: printed parts treated with extraction protocol; C: printed parts treated and then coated with Sylgard-184; D: printed parts cleaned with isopropanol and coated with Sylgard-184) observed at 24, 48, and 72 hours. Fluorescent microscopy images show cell growth around the samples.

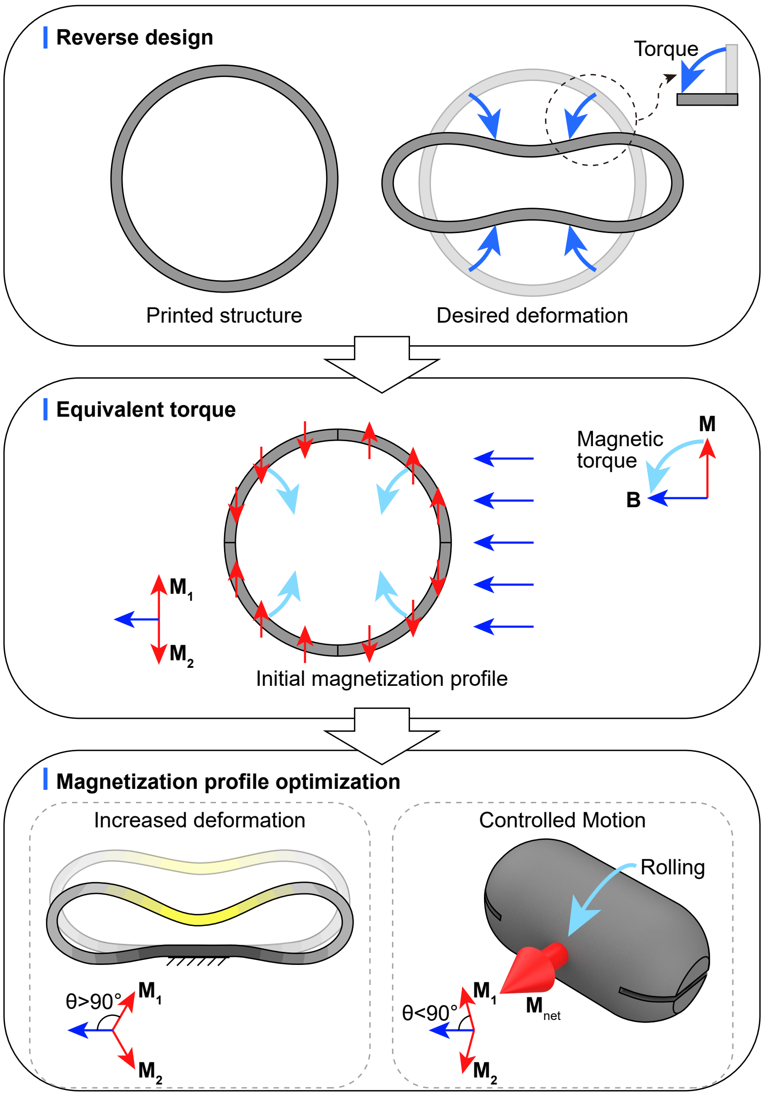

Supplementary Figure 15 Schematic illustration of the function-based inverse design strategy for magnetization profile design. The process consists of three main steps: (1) Reverse design: defining the desired deformation based on the printed structure; (2) Equivalent torque: deriving the initial magnetization profile based on the required magnetic torque; and (3) Magnetization profile optimization: refining the profile for increased deformation or controlled motion.

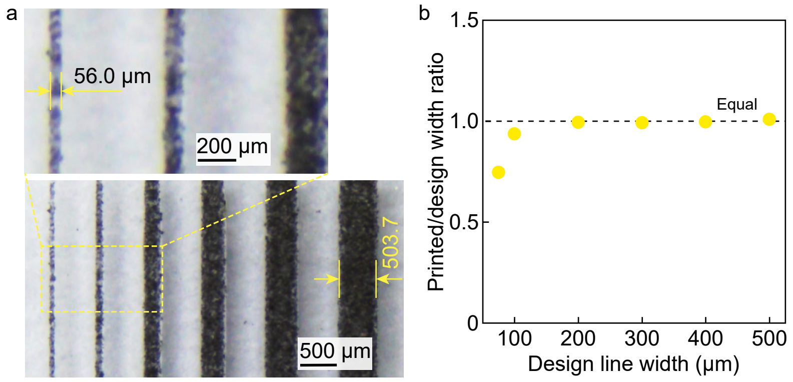

Supplementary Figure 16 Analysis of DLP printing resolution. (a) Microscopic images of printed lines. Top: Magnified view showing detailed width measurement. Bottom: Wider view of multiple lines with varying widths. (b) Printed vs. design line width ratio. Dashed line indicates 1:1 ratio.

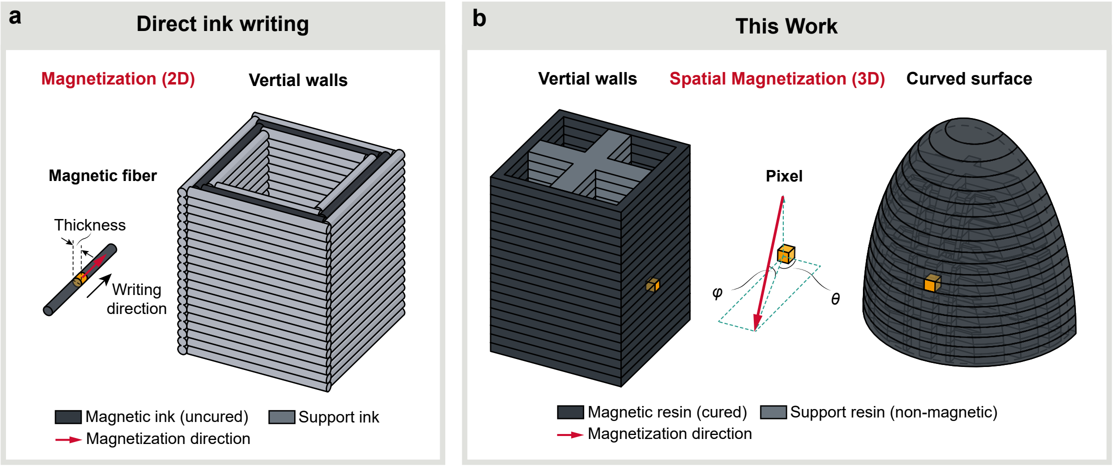

Supplementary Figure 17 Comparison of printing complexity and magnetization control in 3D thin-walled magnetic soft structures (a) 2D tangential magnetization in vertically printed thin-walled structures (Kim et al., *Nature* 2018). (b) 3D spatial magnetization in vertically printed and curved thin-walled structures enabled by DLP printing (this work).

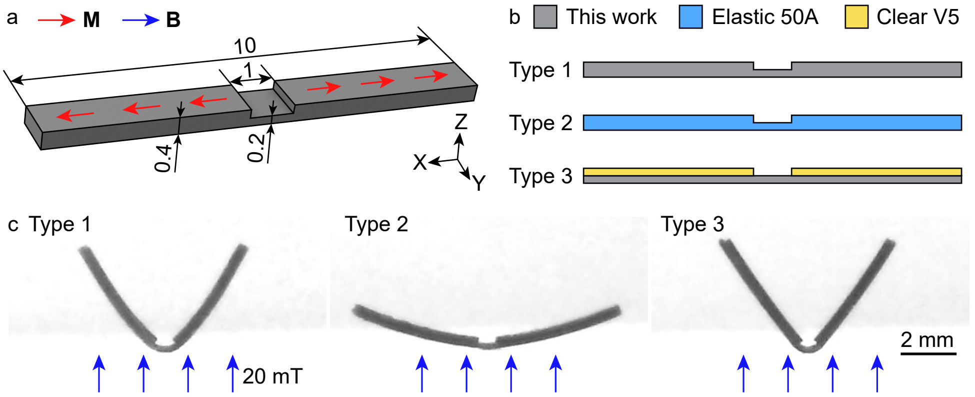

Supplementary Figure 18 Material compatibility and multi-material printing demonstration. (a) Schematic of the hinged rectangular flap with dimensions (in mm) and magnetization direction (**M**, red arrows) relative to the applied magnetic field (**B**, blue arrows). (b) Cross-sectional view of three types of printed structures using different materials: Type 1 (This work, gray) made entirely of our photocurable PDMS, Type 2 (Elastic 50A, blue) made of commercial flexible resin, and Type 3 (combination of This work and Clear V5, yellow) demonstrating multi-material printing. (c) Deformation of the three types under a 20 mT external magnetic field, showing distinct bending behaviors: Type 1 forms a narrow 'V' shape, Type 2 exhibits minimal bending, and Type 3 forms a wider 'V' shape due to the rigid upper arms.

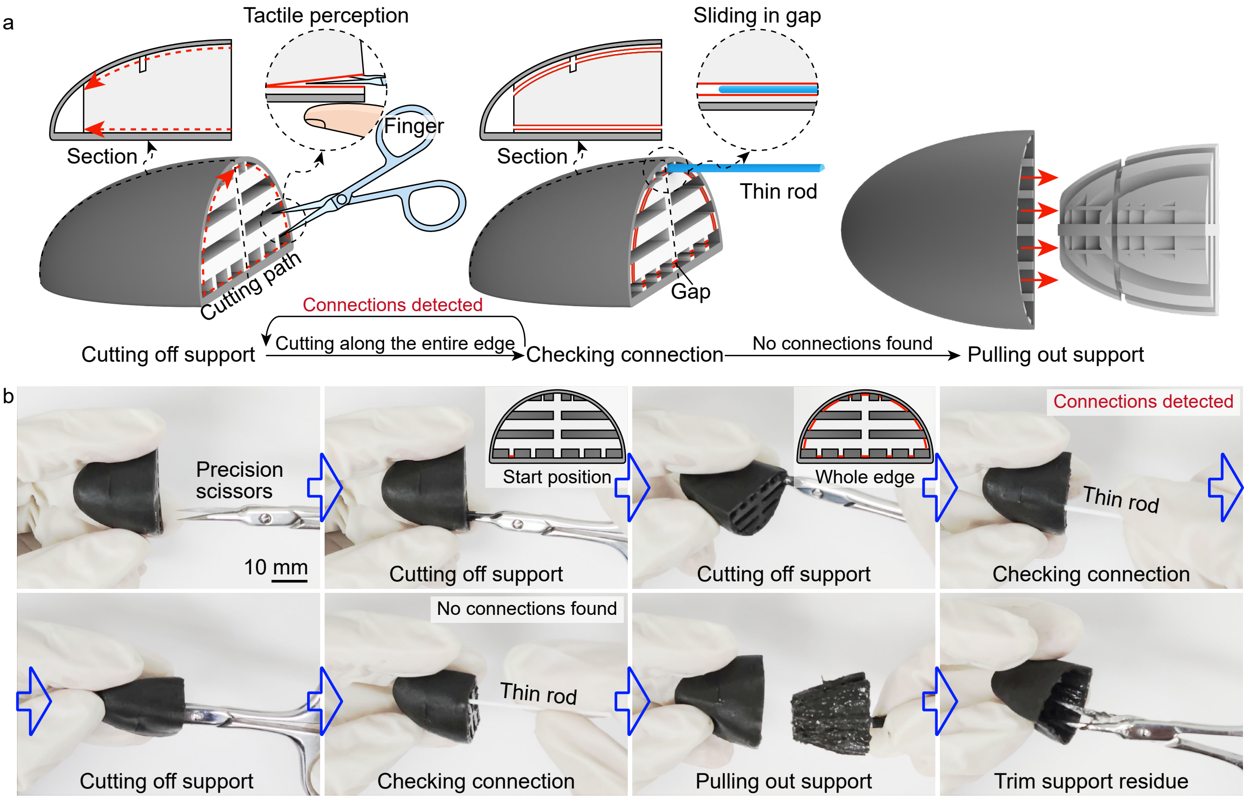

Supplementary Figure 19 Detailed illustration and experimental demonstration of the support structure removal process. (a) Schematic diagram showing the three key steps of support removal: cutting off support, checking connection, and pulling out support. The process utilizes tactile perception and a thin rod for precise control. (b) Experiment images of the actual support removal process for a complex 3D printed structure. The sequence demonstrates the use of precision scissors, the cutting process along the whole edge, connection checking with a thin rod, and final removal and trimming of support residues.

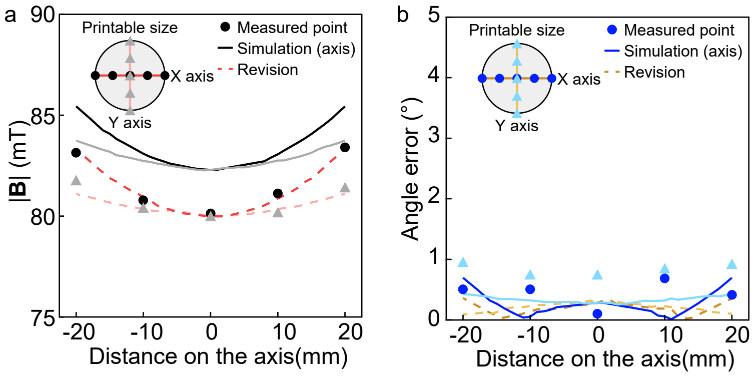

Supplementary Figure 20 Comparison of measured, simulated, and revised simulation data for magnetic field characteristics. (a) Magnetic field strength |**B**| and (b) Angle error as a function of distance along the X and Y axes within the printable size. Black dots and blue dots represent measured points for field strength and angle error, respectively. Solid lines show the original simulation results, while dashed lines indicate the revised simulation after adjusting the remanent flux density. The circular insets illustrate the printable area and measurement axes.

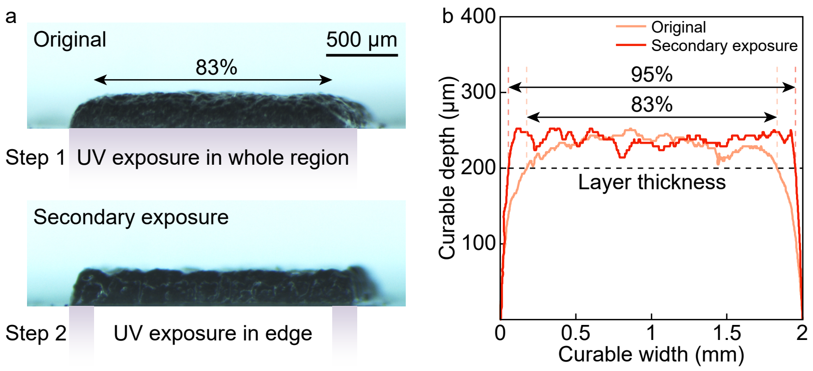

Supplementary Figure 21 Improvement of layer thickness uniformity through secondary exposure. (a) Side-wall images of cured structures showing the original single exposure result (top) and the improved result after secondary exposure (bottom). The width of the cured region increases from 83% to 95% of the projected area width after secondary exposure. (b) Curable depth profiles across the width of the projected area. The original single exposure (orange line) shows non-uniformity, with the edge regions having a lower curable depth. After secondary exposure (red line), the uniformity is greatly improved, with the curable depth at the edges increasing to match that of the central region more closely. The dashed line indicates the target layer thickness

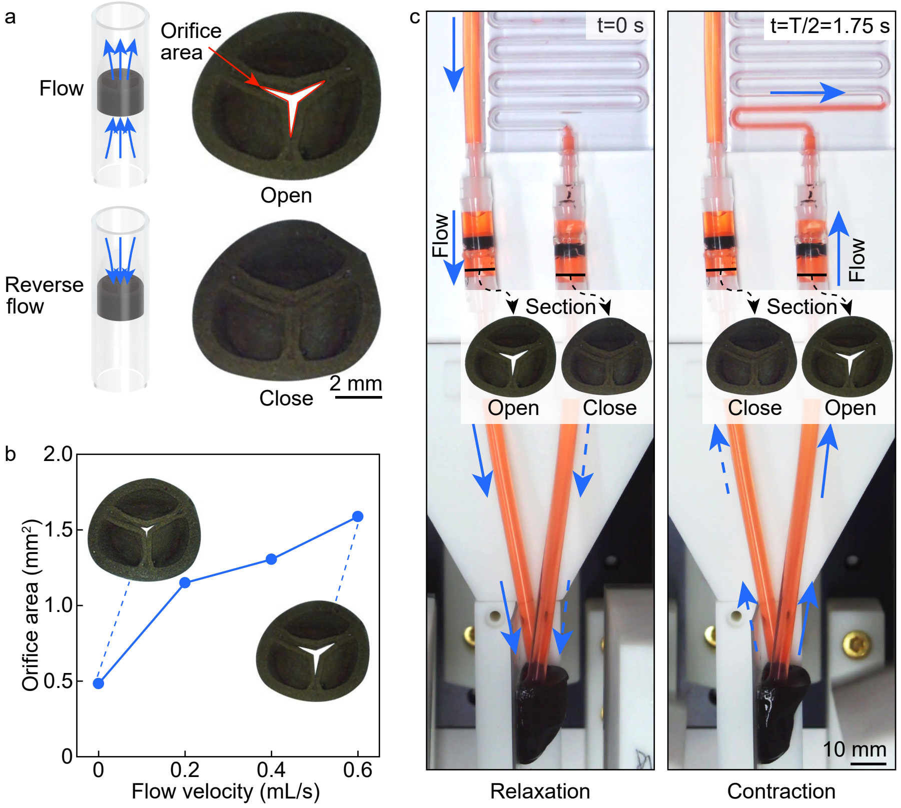

Supplementary Figure 22 Fluid pressure-controlled unidirectional valve system inspired by heart and its working principle. (a) Open and close states of the valve under forward and reverse flow. (b) Relationship between flow velocity and valve orifice area. (c) Coordinated operation of two valves during a complete pumping cycle.

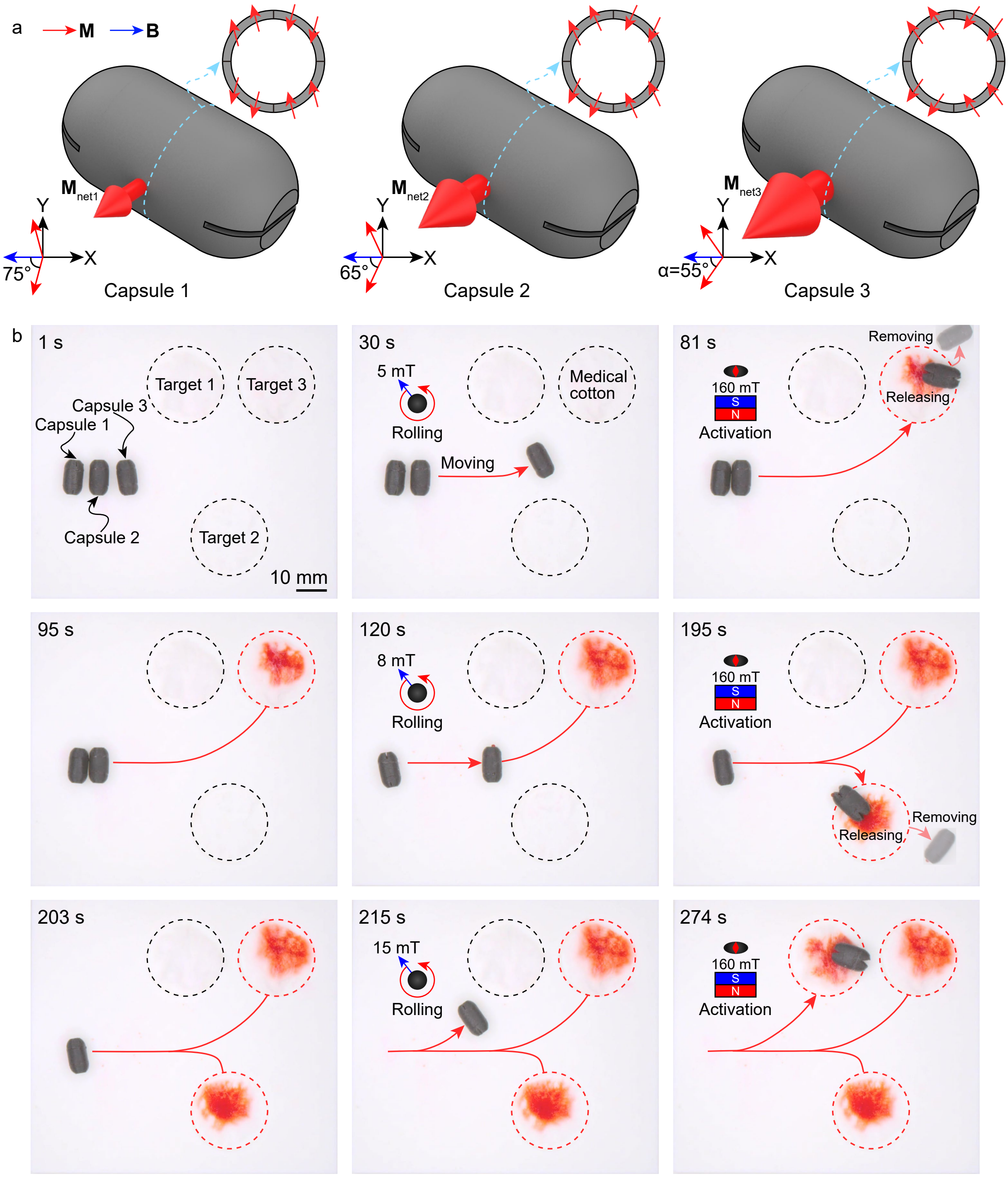

Supplementary Figure 23 Magnetization-programmed soft capsules for multi-region targeted delivery. (a) Schematic of three soft capsules with different magnetization angles and resulting net magnetic moments. (b) Time-lapse sequence demonstrating independent control and sequential payload release of three magnetization-programmed soft capsules under varying magnetic field strengths.

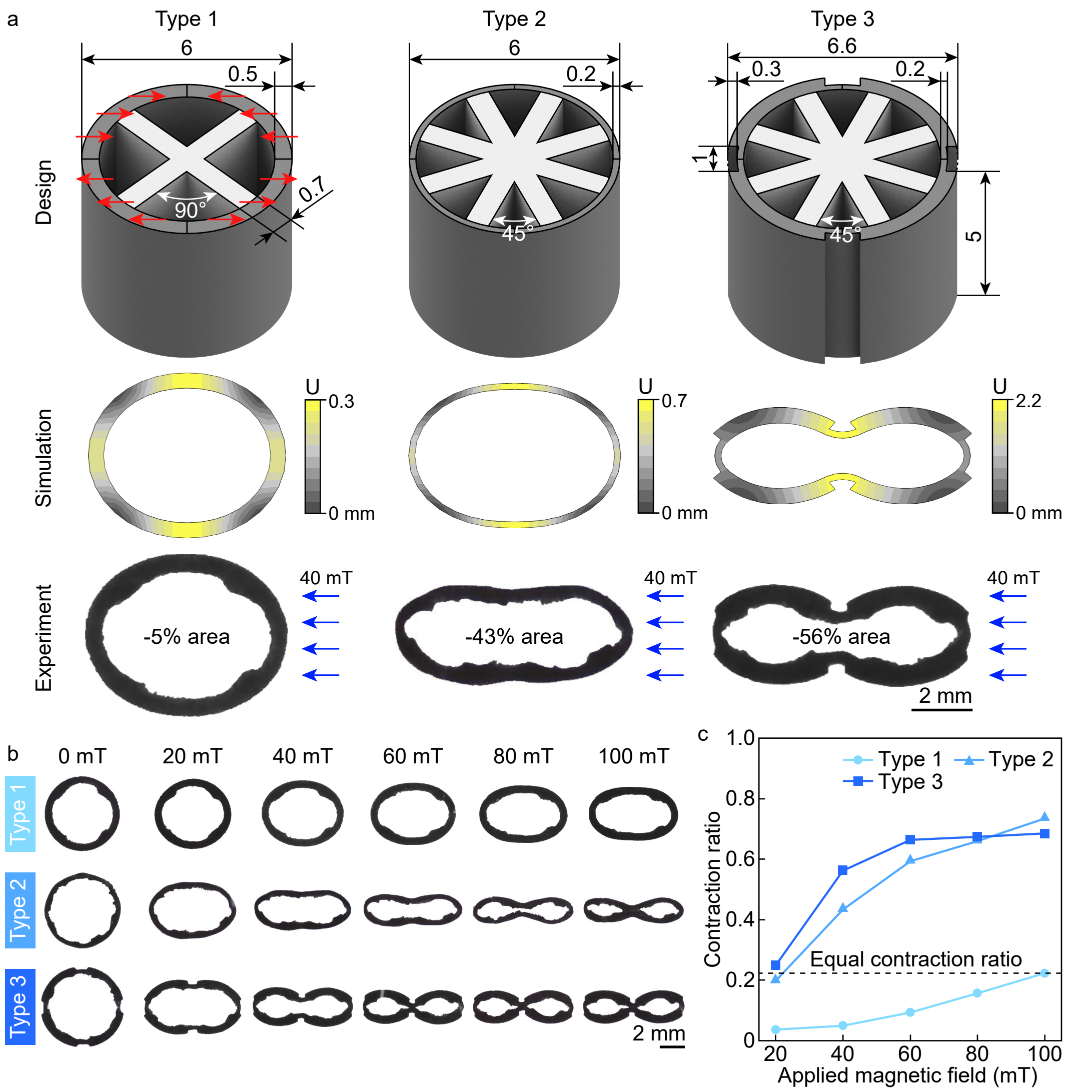

Supplementary Figure 24 Structural design optimization and performance comparison of magnetically responsive 3D printed structures. (a) Design, simulation, and experimental results for three types of cylindrical structures under a 40 mT magnetic field. Type 1: uniform wall thickness; Type 2: reduced wall thickness; Type 3: optimized wall thickness distribution. (b) Cross-sectional deformation of the three structure types under increasing magnetic field strengths from 0 to 100 mT. (c) Contraction ratio analysis of the three structure types as a function of applied magnetic field strength.

Supplementary Table

Supplementary Table 1 Fabrication and support removal details of all prototyped soft devices.

Prototyped Devices	Batches	Units/ Batch	Layers/ Unit	Mag. Dir./ Layer	Time/ Batch (hrs)	Total Time (hrs)	Supp. Rem./ Unit (mins)	Hollow Ratios
Cube	1	1	50	2∼5	5.1	5.1	0	N/A
Thin-walled tube	1	1	50	2	2.4	2.4	5	0.92
Biomimetic aortic valve	1	1	15	3	0.9	0.9	0	N/A
Biomimetic mitral valve	1	1	25	2	1.2	1.2	2	N/A
Octagonal tube	1	1	50	2	2.4	2.4	5	0.72
Multi-unit 3D lattice	1	1	15	2	0.7	0.7	5	0.53
Biomimetic colonic peristaltic machine	2	2	50	2	2.4	4.8	5	0.81
Biomimetic heart pumping machine	1	1	121	2	3.2	3.2	10	0.81
Capsule Robots	1	6	55	2	2.6	2.6	5	0.65

* **Batches:** Number of print batches. **Units/Batch:** Number of units printed per batch. **Layers/Unit:** Number of layers for each unit. **Mag. Dir./Layer:** Number of different magnetization field directions applied per layer. **Time/Batch (hrs):** Fabrication time required for each batch in hours. **Total Time (hrs):** Total fabrication time for all batches combined. **Supp. Rem./Unit (mins):** Time required to remove support structures for a single unit. **Hollow Ratio:** Ratio of internal cavity volume (V_{hollow}) to total volume of the device (V_{total}), calculated as $V_{\text{hollow}}/V_{\text{total}}$. **N/A** indicates that the hollow ratio is not applicable due to the lack of internal cavities.

References

[1] Halbach, K.: Design of permanent multipole magnets with oriented rare earth cobalt material. Nuclear instruments and methods **169**(1), 1–10 (1980)

[2] Soltner, H., Blümler, P.: Dipolar halbach magnet stacks made from identically shaped permanent magnets for magnetic resonance. Concepts in magnetic resonance part a **36**(4), 211–222 (2010)

[3] Bhattacharjee, N., Parra-Cabrera, C., Kim, Y.T., Kuo, A.P., Folch, A.: Desktop-stereolithography 3d-printing of a poly (dimethylsiloxane)-based material with sylgard-184 properties. Advanced materials **30**(22), 1800001 (2018)

[4] Wang, Y., Wang, Y., Mao, C., Mei, D.: Printing depth modeling, printing process quantification and quick-decision of printing parameters in micro-vat polymerization. Materials & Design **227**, 111698 (2023)

[5] Xue, D., Wang, Y., Mei, D.: Multi-step exposure method for improving structure flatness in digital light processing-based printing. Journal of Manufacturing Processes **39**, 106–113 (2019)
