# Supplementary material for: Programmable spatial magnetization stereolithographic printing of biomimetic soft machines with thin-walled structures
Source: Nat Commun. 2024 Nov 30;15:10442. doi: 10.1038/s41467-024-54773-2 (PMC11608252; doi:10.1038/s41467-024-54773-2)
Supplement: Supplementary file 2 — Description of Additional Supplementary Files [file 41467_2024_54773_MOESM2_ESM.pdf]

<u>Description of Additional Supplementary Files</u>

Supplementary Movie 1.mp4 (Biomimetic colonic machine mimicking the intestine's peristaltic defecation)

Supplementary Movie 2.mp4 (Biomimetic heart machine pumping fluid by cyclic diastole and systole)

Supplementary Movie 3.mp4 (All test experiments of the soft capsule robots with potential biomedical functionalities)

Supplementary Movie 4.mp4 (Capsule robots' team achieves targeted drug delivery in multiple regions)
